# QTOF-ESI MS Characterization and Antioxidant Activity of *Physalis peruviana* L. (Cape Gooseberry) Husks and Fruits from Costa Rica

**DOI:** 10.3390/molecules27134238

**Published:** 2022-06-30

**Authors:** Mirtha Navarro-Hoyos, Elizabeth Arnáez-Serrano, María Isabel Quirós-Fallas, Felipe Vargas-Huertas, Krissia Wilhelm-Romero, Felipe Vásquez-Castro, Diego Alvarado-Corella, Andrés Sánchez-Kopper

**Affiliations:** 1 Bioactivity and Sustainable Development (BIODESS) Group, Department of Chemistry, University of Costa Rica (UCR), San Jose 2060, Costa Rica; maria.quirosfallas@ucr.ac.cr (M.I.Q.-F.); luis.vargashuertas@ucr.ac.cr (F.V.-H.); krissia.wilhelm@ucr.ac.cr (K.W.-R.); manuel.vasquezcastro@ucr.ac.cr (F.V.-C.); luis.alvaradocorella@ucr.ac.cr (D.A.-C.); 2 Department of Biology, Costa Rica Institute of Technology (TEC), Cartago 7050, Costa Rica; earnaez@itcr.ac.cr; 3 Centro de Investigación y de Servicios Químicos y Microbiológicos (CEQIATEC), Department of Chemistry, Costa Rica Institute of Technology (TEC), Cartago 7050, Costa Rica; ansanchez@itcr.ac.cr

**Keywords:** *Physalis peruviana*, withanolides, flavonoids, sucroses, UPLC-DAD, QTOF-ESI MS

## Abstract

There is increasing interest in research of secondary metabolites from *Physalis peruviana* (Cape gooseberry) because of their potential bioactivities. In this study, the profile of compounds found in fruits and husks from Costa Rica was determined through ultra-performance liquid chromatography coupled with high-resolution mass spectrometry using a quadrupole time-of-flight analyzer (UPLC-ESI-QTOF MS) on extracts (*n* = 10) obtained through pressurized liquid extraction (PLE) conditions. In total, 66 different compounds were identified, comprising 34 withanolides, 23 sucrose ester derivatives and 9 flavonoids. UPLC-DAD analysis was performed to determine the β-carotene in fruits and to quantify the flavonoids in all 10 samples, with the results showing higher contents in samples from the Dota region (58.6–60.1 μg/g of dry material versus 1.6–2.8 mg/g of dry material). The Folin–Ciocalteau total polyphenolic content (FC) and antioxidant activity using the DPPH method showed better results for the husk extracts, with the ones from the Dota region holding the best values (4.3–5.1 mg GAE/g of dry material versus IC_50_ = 1.6–2.3 mg of dry material/mL). In addition, a significant negative correlation was found between the RU, FC and DPPH values (r = −0.902, *p* < 0.05), aligning with previous reports on the role of polyphenols in antioxidant activity. Principal correlation analysis (PCoA) and hierarchical clustering (HC) analysis were performed on HRMS results, and they indicated that the D1 and D2 fruit samples from the Dota region were clustered with husks related to a higher presence of the analyzed metabolites. In turn, principal component analysis (PCA) performed on the flavonoid content and antioxidant activity yielded results indicating that the D1 and D2 husks and fruit samples from the Dota region stood out significantly, showing the highest antioxidant activity. In summation, our findings suggest that *P. peruviana* husks and fruits from Costa Rica constitute a substrate of interest for further studies on their potential health benefits.

## 1. Introduction

Cape gooseberry (*Physalis peruviana* L.), also commonly known as goldenberry, is a member of the Solanaceae family which is native to the Andes and has been introduced in different countries since its domestication [1]. Antispasmodic, diuretic, antiseptic, sedative and analgesic properties, in addition to throat trouble relief and the elimination of intestinal parasites and amoeba, are among the properties attributed to *P. peruviana* fruits and husks [2,3], and they are used empirically in traditional medicine to treat cancer and other diseases such as hepatitis, asthma, malaria and dermatitis [4] or as antimicrobial, antipyretic, diuretic or anti-inflammatory immunomodulators [5].

Some of these medicinal properties have been studied through in vitro methodologies, with the extracts showing relevant antioxidant [6], antimicrobial [7], anti-inflammatory [8], cytotoxic [9], anti-hepatotoxic [10], antiproliferative [11], immunomodulatory [12] and anti-glycemic [13] activities. These properties are known to be related to the role of secondary metabolite bioactivities [14].

Withanolides present in *P. peruviana* represent a collection of naturally occurring C-28 steroidal lactone triterpenoids assembled on an integral or reorganized ergostane structure, in which C-22 and C-26 are oxidized to form a six-membered lactone ring [15]. These molecules account for properties such as anti-tumor [16], anti-arthritic [17], anti-aging [18], neuroprotective [19] and hypercholesteremia suppression activity [20], and there is also evidence of their immune modulatory potential [21]. *P. peruviana* fruit is also a natural source of β-carotene [22] sought after for its antioxidant potential [23], its ability to be converted into vitamin A [24] and for helping prevent heart disease by blocking the formation and oxidation of low-density lipoproteins [25].

Flavonoids have also been reported in *P. peruviana* fruits and husks [26]. Among these are quercetin and its derivatives, which show biological activities dependent on modifications in the molecule [27,28]. For instance, rutin is a quercetin derivative with a rutinoside glycosylation at the 3-O position, with anticonvulsant, antidepressant, analgesic, antidiabetic and antiasthmatic properties and wound-healing effects [29]. Finally, another bioactive group found in *P. peruviana* fruits and husks includes functionalized sucroses [26], which are metabolites that present anti-inflammatory activity [30] and have been reported as α-amylase inhibitors, explaining their traditional use as hypoglycemic agents [31].

The present work addresses the UPLC-ESI-QTOF MS characterization of the withanolides, functionalized sucroses and flavonoids present in *P. peruviana* extracts (*n* = 10) from fruits and husks purchased from the main local producers located in the Dota and Paraiso regions in Costa Rica. In addition, UPLC-DAD determination of β-carotene and quantification of flavonoids were performed, as well as evaluation of the FC total polyphenolic contents and DPPH antioxidant scavenging activity. Finally, statistical analyses on the HRMS results and on the flavonoid contents and antioxidant values were performed. This work constitutes, to our knowledge, the first study on *P. peruviana* (Cape gooseberry) husks and fruits from Central America.

## 2. Results and Discussion

### 2.1. P. peruviana Husk and Fruit Secondary Metabolite Profile by UPLC-ESI-MS Analysis

The UPLC-QTOF-ESI MS analysis described in the Materials and Methods section allowed us to identify 66 compunds previously reported in *P. peruviana* fruits and husks, including 34 withanolide derivatives, 23 sucrose derivatives and 9 flavonoids. Table S1 (Supplementary Materials) summarizes the results for the husks and fruits from the Dota and Paraiso regions in Costa Rica (*n* = 10).

#### 2.1.1. Withanolides

**Figure 1:**
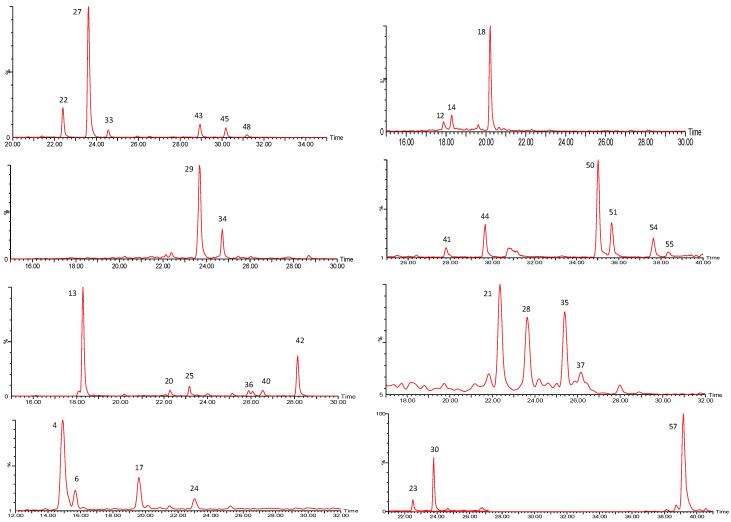
UPLC QTOF-ESI MS extracted ion chromatograms of withanolides from *P. peruviana* husks and fruits.

The first group of compounds identified in the *P. peruviana* samples was constituted by whitanolides, which are C-28 steroids based on the ergostane skeleton functionalized at C-22 and C-26 with δ-lactone rings. Figure 1 shows the chromatograms of the 34 withanolides found in the samples.

**Figure 2:**
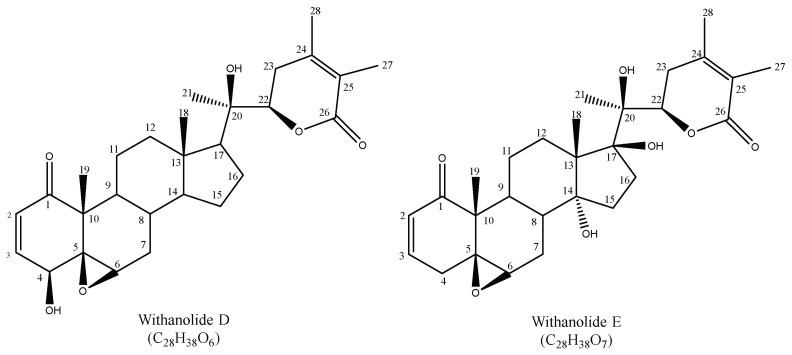
Withanolide D and E structures.

The derivatives from withanolides D and E (Figure 2), important backbones for withanolides [32,33,34] found in the husks and fruits of *P. peruviana* from Costa Rica, include 34 previously described compounds, as shown in Table S1 (Supplementary Materials).

Table 1 summarizes these compounds’ molecular ion [M-H]^−^ and the common fragmentation pattern due to the loss of their lactone moieties [M-H-Lac]^−^ and the loss of water molecules [M-H-H_2_O]^−^ and [M-H-2H_2_O]^−^, as well as fragments derived from the loss of their lactone moiety and water altogether [M-H-Lac-H_2_O]^−^ and [M-H-Lac-2H_2_O]^−^. Other observed fragments corresponded with the loss of acetyl, methoxy or glycoside moieties if present in the compound [26].

**Figure 3:**
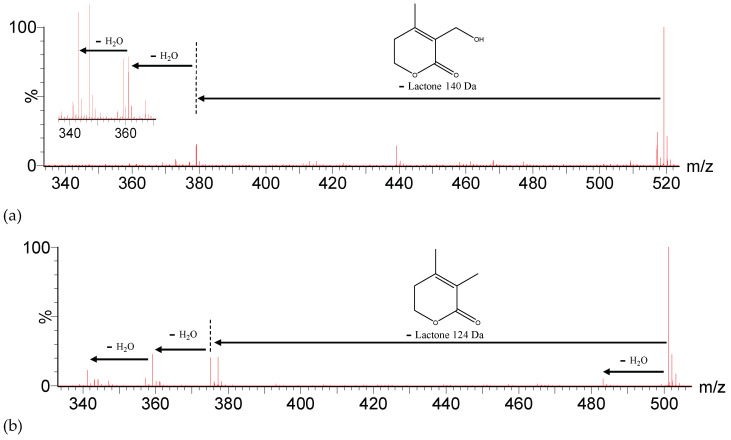
Fragmentation ion (MS^2^) spectra for (**a**) compound 13, [M-H]^−^ = 519.2601, with enlargement of range *m*/*z* 335–370, and (**b**) compound 27, [M-H]^−^ = 501.2494.

For instance, compound 13 ([M-H]^−^ = 519.2601), tentatively assigned to the previously described *2,3-dihydro-4,27-dihydroxylated withanolide E* [26], exhibited a predominant fragment at *m*/*z* 379, corresponding to the loss of the lactone moiety with a mass of 140 Da (Figure 3a), with other fragments corresponding to the loss of water, exhibiting lower intensities. Meanwhile, compound 27 ([M-H]^−^ = 501.2494), tentatively identified as the previously reported *17,27-dihydroxylated withanolide D* [26], exhibited a loss of lactone moiety of 124 Da in a fragment at *m*/*z* 377, and the predominant fragment at *m*/*z* 359 was generated from a loss of lactone moiety of 124 Da and one water molecule [M-H-Lac-H_2_O]^−^ (Figure 3b). Furthermore, it has been previously reported that the successive loss of water molecules from the ergostane moiety is consistent with the presence of a hydroxyl in C4 and the epoxide in C5–C6, forming an extended conjugation with the double bond in C2–C3 [26,35].

**Figure 4:**
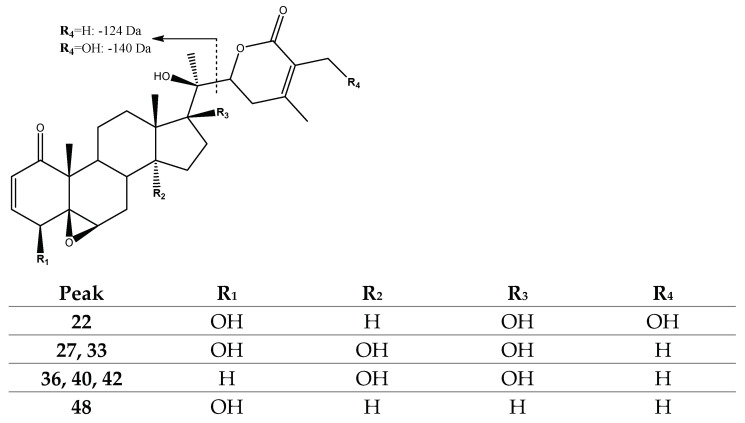
Structure and fragmentation of withanolide D derivatives 22 and 48.

Aside from peak 27 with a retention time (Rt) at 23.57 min, peak 33 (Rt = 24.56 min) was also tentatively assigned to the previously described *4β-hydroxywithanolide E* isomer [26], while other withanolide D and E derivatives previously reported [26] were present in peak 48 (Rt = 31.19 min) with [M-H]^−^ at *m*/*z* 469.2607 (C_28_H_37_O_6_) and peaks 36 (Rt = 25.89 min), 40 (Rt = 26.55 min) and 42 (Rt = 28.15 min) present with [M-H]^−^ at *m*/*z* 485.2550 (C_28_H_37_O_7_) and with fragments at *m*/*z* 345 and *m*/*z* 361 [M-H-124]^−^, respectively, corresponding to the loss of the lactone residue (124 Da). In turn, peak 22 (Rt = 22.38 min), with [M-H]^−^ at *m*/*z* 501.2494 (C_28_H_37_O_8_) and tentatively assigned to the previously described *17,27-dihydroxylated withanolide D* [26], exhibited a loss of the C-27 hydroxylated lactone moiety (140 Da) at *m*/*z* 361 [M-H-140]^−^ (Figure 4).

**Figure 5:**
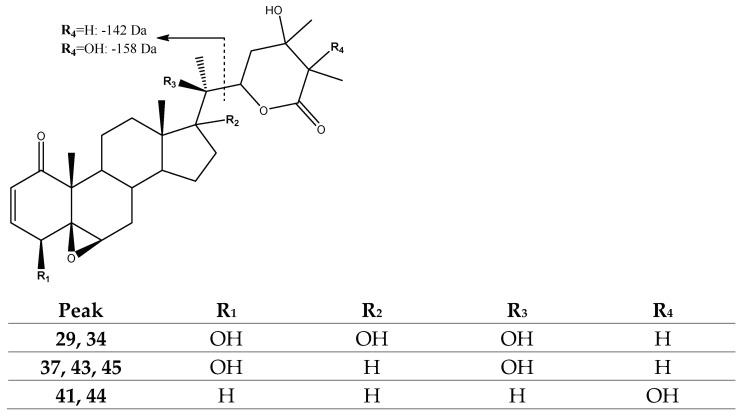
Structure and fragmentation of withanolide D derivatives with hydrogenated lactone.

Peaks 37 (Rt = 25.93 min), 43 (Rt = 28.94 min) and 45 (Rt = 30.18 min), with [M-H]^−^ at *m*/*z* 487.2701 (C_28_H_39_O_7_), which were tentatively identified as previously described *24,25-dihydro-24-hydroxylated withanolide D* derivatives [26], and peaks 29 (Rt = 23.64 min) and 34 (Rt = 24.72 min), with [M-H]^−^ at *m*/*z* 503.2650 (C_28_H_39_O_8_), which were tentatively assigned to previously reported *24,25-dihydro-17,24-dihydroxylated withanolide D* derivatives [26], showed the loss of hydroxylated lactone hydrated at C24-C25 (142 Da) in fragments [M-H-142]^−^ at *m*/*z* 345 and *m*/*z* 361, respectively. In turn, peaks 41 (Rt = 27.81) and 44 (Rt = 29.67) with [M-H]^−^ at *m*/*z* 471.2725 (C_28_H_39_O_6_), tentatively identified as previously reported *Peruvianolide D* isomers [36], showed a loss of dihydroxylated lactone (158 Da), exhibiting a fragment at *m*/*z* 313 [M-H-158]^−^ (Figure 5).

**Figure 6:**
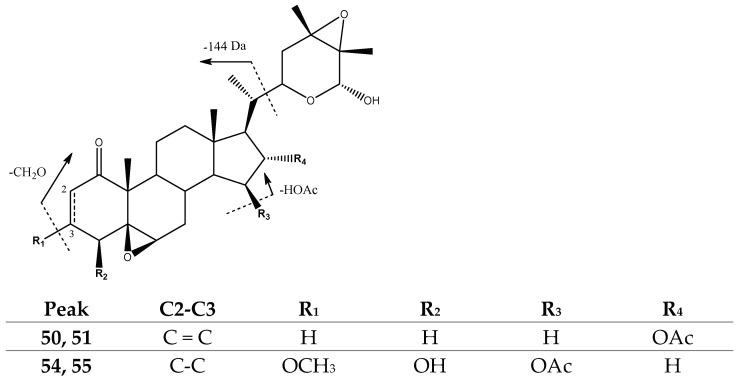
Structure and fragmentation of Virginol C and Peruvianolide E isomers.

Peaks 50 (Rt = 35.02 min) and 51 (Rt = 35.66 min), tentatively assigned to previously described *Virginol C* isomers [37] with [M-H]^−^ at *m*/*z* 513.2831 (C_30_H_41_O_7_), and peaks 54 (Rt = 37.65 min) and 55 (Rt = 38.31 min), tentatively assigned to previously reported *Peruvianolide E* isomers [36] with [M-H]^−^ = 561.3042 (C_31_H_45_O_9_), showed a loss of the acetoxy group (−60 Da) and neutral loss of the 6-member ring with an epoxide (144 Da), yielding their main fragments at *m*/*z* 309 [M-H-HOAc-Lac]^−^ and *m*/*z* 291, respectively. This last aspect was due to the additional loss of the methoxy group in compounds 54 and 55 (Figure 6).

**Figure 7:**
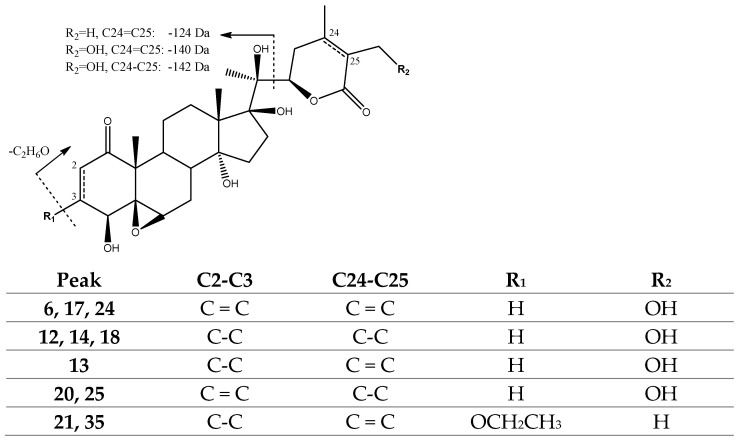
Structure and fragmentation of withanolide E derivatives hydroxylated in C-27.

Peaks 6 (Rt = 15.68 min), 17 (Rt = 19.60 min) and 24 (Rt = 23.01 min), with [M-H]^−^ at *m*/*z* 517.2471 (C_28_H_37_O_9_), were tentatively assigned to previously described *4β,27-dihydroxywithanolide E isomers* [26], with the loss of the hydroxylated lactone yielding a fragment at *m*/*z* 377 [M-H-140]^−^. In turn, peaks 20 (Rt = 22.28 min) and 25 (Rt = 23.19 min), with [M-H]^−^ at *m*/*z* at 519.2601 (C_28_H_39_O_9_), and peaks 12 (Rt = 17.88 min), 14 (Rt = 18.30 min) and 18 (Rt = 20.22 min), with [M-H]^−^ at *m*/*z* 521.2756 (C_28_H_41_O_9_), were tentatively assigned to previously described *24,25-Dihydro-4,27-dihydroxylated withanolide E* and *2,3,24,25-tetrahydro-4,27-dihydroxylated withanolide E* isomers [26], with the loss of hydrogenated lactone moiety (142 Da) yielding fragments at *m*/*z* 377 and *m*/*z* 379 [M-H-142]^−^, respectively. Finally, peaks 21 (Rt = 22.33 min) and 35 (Rt = 25.38 min) were tentatively assigned to previously described *Phyperunolide F* isomers [38], with [M-H]^−^ at *m*/*z* 547.2926 (C_30_H_43_O_9_) and fragments at *m*/*z* 501 [M-H-C_2_H_6_O]^−^ due to the loss of ethanol moiety and *m*/*z* 341 due to the loss of lactone (−124 Da) and two molecules of water (Figure 7).

**Figure 8:**
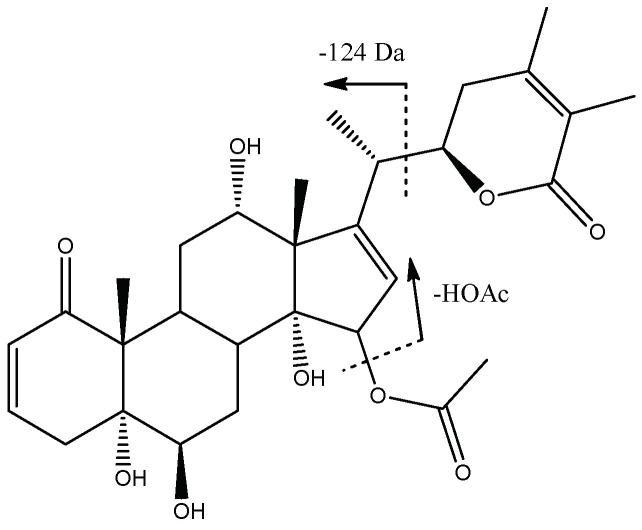
Structure and fragmentation of Physagulin O.

Another type of withanolide E was present in peak 4 (Rt = 14.91 min) with [M-H]^−^ at *m*/*z* 543.2612 (C_30_H_39_O_9_), which was tentatively assigned to the previously described Physagulin O [32] (Figure 8), with fragments at *m*/*z* 483 due to the loss of the acetoxy group (−60 Da) and *m*/*z* 359 from the loss of lactone (124 Da).

**Figure 9:**
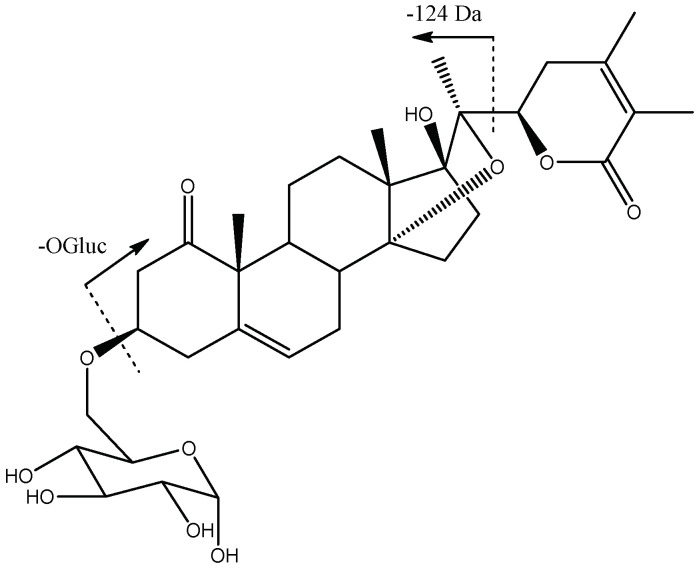
14,20-Epoxy-(22R)-3β-(O-β-glucopyranosyl)-17β-hydroxy-1-oxowitha-5,24-dienolide structure and fragmentation.

Peak 28 (Rt = 23.59 min) with [M-H]^−^ at *m*/*z* 631.3137 (C_34_H_47_O_11_) was tentatively assigned to the previously described *14,20-Epoxy-(22R)-3β-(O-β-glucopyranosyl)-17β-hydroxy-1-oxowitha-5,24-dienolide* [39], with fragments at *m*/*z* 451 due the loss of glucose [M-H-OGluc]^−^ and *m*/*z* 433 [M-H-OGluc-H_2_O]^−^ and *m*/*z* 309 [M-H-Gluc-H_2_O-124]^−^ due to the loss of lactone (124 Da), as shown in Figure 9.

**Figure 10:**
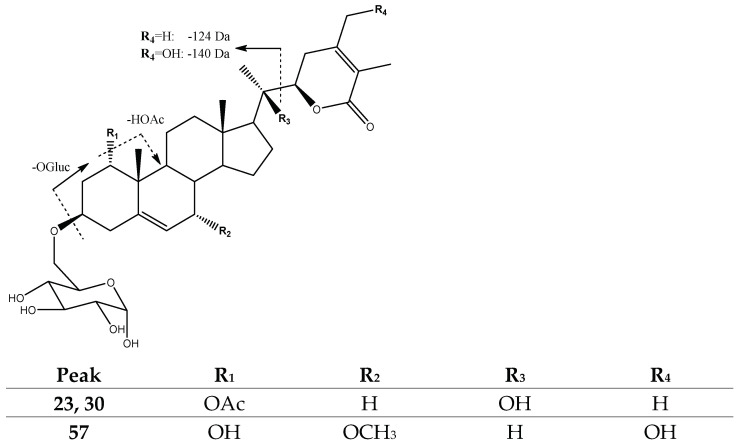
Structure and main fragments of Physalolactone B-3-O-β-glucopyranoside isomers (23 and 30) and Daturafoliside B (57).

Finally, peaks 23 (Rt = 22.51 min) and 30 (Rt = 23.66 min), with [M-H]^−^ at *m*/*z* 661.3590 (C_36_H_53_O_11_), and peak 57 (Rt = 39.17 min), with [M-H]^−^ at *m*/*z* 649.3606 (C_35_H_53_O_11_), were tentatively assigned to previously described *Physalolactone B-3-O-β-glucopyranoside* isomers [32] and *Daturafosilide B* [40], with a common fragmentation pattern due to the loss of glucose-yielding fragments at *m*/*z* 481 and *m*/*z* 469 [M-H-OGluc]^−^, respectively, and the loss of their lactone moieties (−124 and −140 Da) yielding fragments at *m*/*z* 357 and *m*/*z* 329, respectively (Figure 10).

#### 2.1.2. Sucrose Ester Derivatives

**Figure 11:**
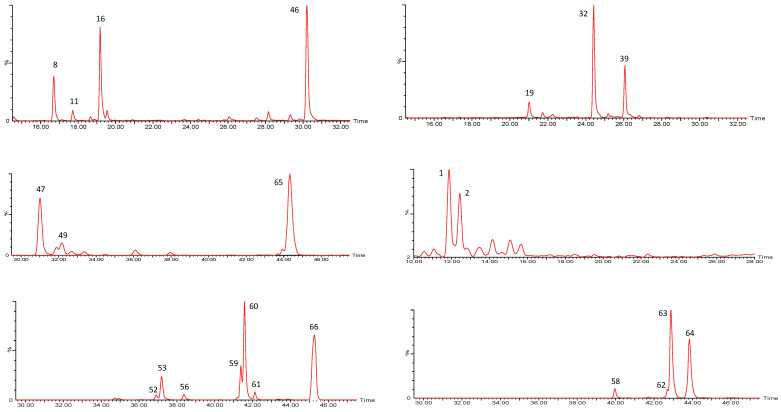
UHPLC QTOF-ESI MS extracted ion chromatograms of sucrose ester derivatives from *P. peruviana* husks and fruits.

The second group of compounds found in the *P. peruviana* samples was constituted by 23 previously described functionalized sucrose esters with isobutanoyl, methylbutanoyl, pentenoyl, octanoyl, nonanoyl, decanoyl and dodecanoyl substituents [26,31], as shown in Table S1 (Supplementary Materials). Figure 11 shows the chromatograms for these 23 compounds, consisting of mono-, di-, tri- and tetrasubstituted sucroses.

Table 2 summarizes the characteristic formic acid adduct [M-H+FA]^−^ for these compounds and the fragmentation pattern consisting of the [M-H]^−^ ion and the initial loss of one R group, such as isobutanoyl (C_4_H_6_O, 70 Da), pentenoyl (C_5_H_6_O, 82 Da), 2-metylbutanoyl (C_5_H_8_O, 84 Da), octanoyl (C_8_H_14_O, 126 Da), nonanoyl (C_9_H_16_O, 140 Da), decanoyl (C_10_H_18_O, 154 Da) and dodecanoyl (C_12_H_22_O, 182 Da), while other observed fragments corresponded to the loss of other R groups or water [26].

**Figure 12:**
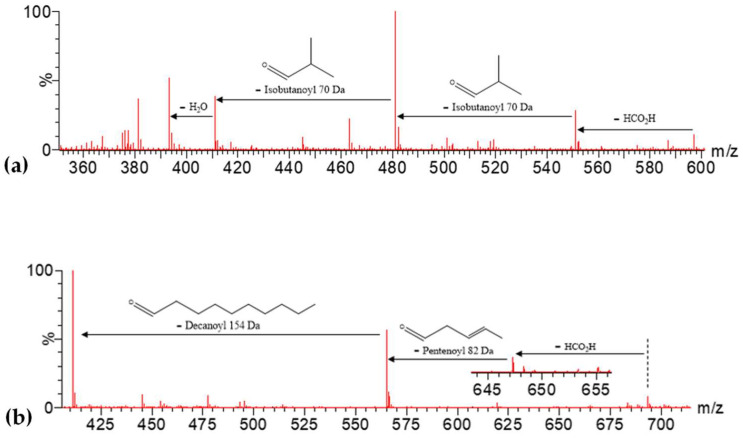
MS^2^ spectra for (**a**) compound 32, [M-H+FA]^−^ = 597.2380, and (**b**) compound 52, [M-H+FA]^−^ = 693.3303, with enlargement for [M-H]^−^ fragment.

For instance, compound 32 ([M-H+FA]^−^ = 597.2380), tentatively assigned to the previously described *tri-O-isobutanoylsucrose* [26], yielded the [M-H]^−^ ion at 551 due to the loss of the formic acid (46 Da) and the main fragment at *m*/*z* 481 [M-H-70]^−^ due to the loss of one isobutanoyl group (70 Da), with other fragments at *m*/*z* 411 [M-H-70-70]^−^ due to the subsequent loss of a second isobutanoyl group and at *m*/*z* 393 due to the subsequent loss of a water molecule (Figure 12a). In turn, compound 52 ([M-H+FA]^−^ = 693.3303), tentatively assigned to the previously described *o-decanoyl-o-isobutanoyl-o-pentenoyl sucrose* [26], showed its main fragments at *m*/*z* 565 [M-H-82]^−^ from the loss of the pentenoyl group (82 Da) and *m*/*z* 411 [M-H-82-154]^−^ due to the subsequent loss of the decanoyl group (154 Da), showing a smaller ion [M-H]^−^ at *m*/*z* 647 (Figure 12b).

**Figure 13:**
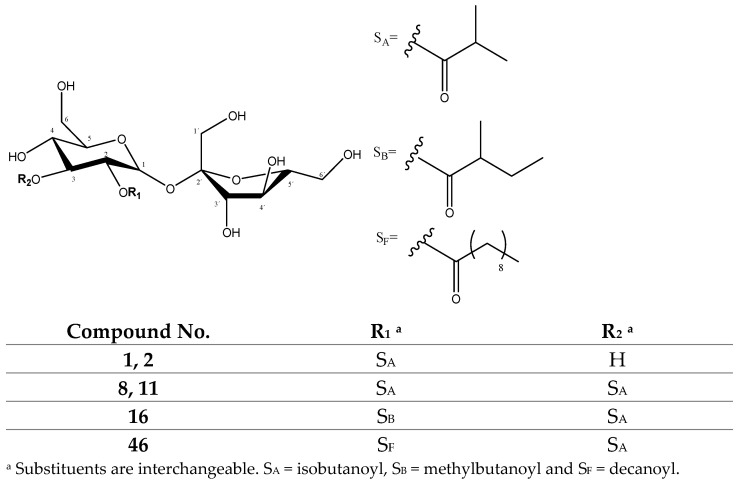
Mono and disubstituted sucrose ester derivatives.

Among the monosubstituted sucroses, peaks 1 (Rt = 11.87 min) and 2 (Rt = 12.43) with [M-H]^−^ at *m*/*z* 411.1528 (C_16_H_27_O_12_) were tentatively assigned to previously described *O-isobutanoylsucrose* isomers [26], with fragments at *m*/*z* 341 [M-H-70]^−^ from the loss of the isobutanoyl group (70 Da). In turn, among the disubstituted sucroses, peaks 8 (Rt = 16.71) and 11 (Rt = 17.72) with [M-H]^−^ at *m*/*z* 481.1960 (C_20_H_33_O_13_), peak 16 (Rt = 19.17 min) with [M-H]^−^ = 495.2117 (C_21_H_35_O_13_) and peak 46 (Rt = 30.21 min) with [M-H]^−^ at 565.2908 (C_26_H_45_O_13_) were tentatively assigned to the previously described *di-O-isobutanoylsucrose, O-isobutanoyl-O-methylbutanoyl sucrose* and *o-decanoyl-o-isobutanoylsucrose* isomers [26], respectively (Figure 13), showing characteristic fragments due to the loss of their R groups (70, 84 and 154 Da, respectively) as shown in Table 2.

**Figure 14:**
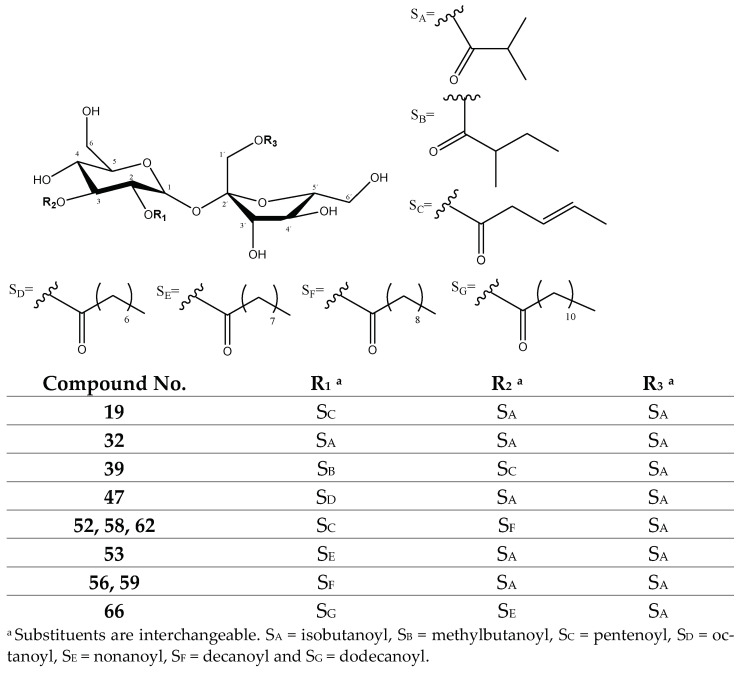
Trisubstituted sucrose ester derivatives.

Among the trisubstituted sucroses, peaks 19 (Rt = 21.04 min) with [M-H]^−^ at *m*/*z* 563.2372 (C_25_H_39_O_14_), 47 (Rt = 31.02 min) with [M-H]^−^ at *m*/*z* 607.3018 (C_28_H_47_O_14_), 32 (Rt = 24.42 min) with [M-H]^−^ = 551.2330 (C_24_H_39_O_14_), 53 (Rt = 37.18 min) with [M-H]^−^ at *m*/*z* 621.3139 (C_29_H_49_O_14_) and 56 (Rt = 38.37 min) and 59 (Rt = 41.44 min) with [M-H]^−^ at *m*/*z* 635.3285 (C_30_H_51_O_14_) constituted compounds with two isobutanoyl groups differing only in the third group and were tentatively assigned to the previously described *tri-O-isobutanoylsucrose, di-o-isobutanoyl-o-pentenoylsucrose*, *di-O-isobutanoyl-O-octanoylsucrose, di-o-isobutanoyl-o-nonanoylsucrose* and *di-o-isobutanoyl-o-decanoylsucrose* isomers [26], respectively (Figure 14), showing characteristic fragments due to the loss of their R groups (70, 82, 126, 140 and 154 Da, respectively) as shown in Table 2.

In turn, peaks 39 (Rt = 26.06 min) with [M-H]^−^ = 577.2532 (C_26_H_41_O_14_) and 66 (Rt = 45.28 min) with [M-H]^−^ = 733.4395 (C_37_H_65_O_14_), as well as peaks 52 (Rt = 36.84 min), 58 (Rt = 39.98 min) and 62 (42.63 min) with [M-H]^−^ at *m*/*z* 647.3217 (C_31_H_51_O_14_), corresponded to compounds with only one isobutanoyl group and were tentatively assigned to previously described *O-isobutanoyl-O-(2-methylbutanoyl)-O-pentenoylsucrose*, *o-dodecanoyl-o-isobutanoyl-o-nonanoylsucrose* and *o-decanoyl-o-isobutanoyl-o-pentenoyl sucrose* isomers [26], respectively (Figure 14), showing characteristic fragments due to the loss of their R groups (70, 82, 84, 140, 154 and 182 Da, respectively) as shown in Table 2.

**Figure 15:**
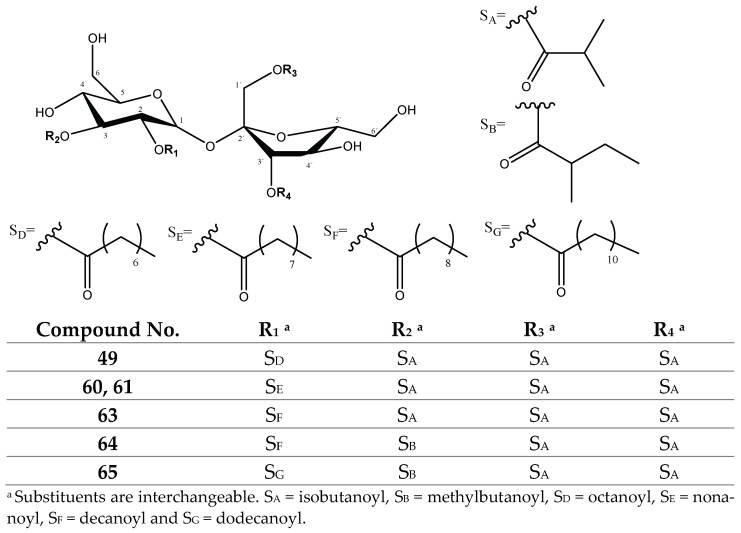
Tetrasubstituted sucrose ester derivatives.

Finally, among the tetrasubstituted sucroses, peaks 49 (Rt = 32.13 min) with [M-H]^−^ = 677.3428 (C_32_H_53_O_15_) and 63 (Rt = 42.89) with [M-H]^−^ ions at *m*/*z* 705.3760 (C_34_H_57_O_15_), as well as peaks 60 (Rt = 41.57 min) and 61 (Rt = 42.11 min) with [M-H]^−^ ions at 691.3513 (C_33_H_55_O_15_), constituted compounds with three isobutanoyl groups differing in only one substituent and were tentatively assigned to the previously described *o-octanoyl-tri-o-isobutanoylsucrose* [31], *o-decanoyl-tri-o-isobutanoylsucrose* [31] and *o-nonanoyl-tri-o-isobutanoylsucrose* isomers [26], respectively (Figure 15), showing characteristic fragments due to the loss of their R groups (70, 126, 140 and 154 Da, respectively) as shown in Table 2.

**Figure 16:**
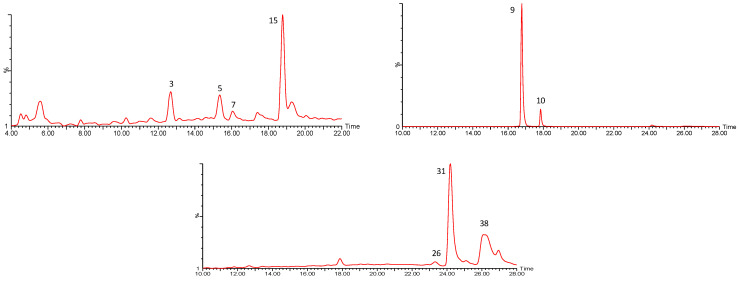
UPLC QTOF-ESI MS extracted ion chromatograms of flavonoids from *P. peruviana* husks and fruits.

Meanwhile, peaks 64 (Rt = 43.85 min) with [M-H]^−^ = 719.3883 (C_35_H_59_O_15_) and 65 (Rt = 44.34 min) with [M-H]^−^ at *m*/*z* 747.4202 (C_37_H_63_O_15_) corresponded to compounds with two isobutanoyl groups and were tentatively assigned to the previously described *di-o-isobutanoyl-o-decanoyl-o-(2-methylbutanoyl) sucrose* and *di-o-isobutanoyl-o-dodecanoyl-o-(2-methylbutanoyl) sucrose* isomers [31], respectively (Figure 16), showing characteristic fragments due to the loss of their R groups (70, 84, 154 and 182 Da, respectively), as shown in Table 2.

#### 2.1.3. Flavonoids

A third group of compounds previously described in *P. peruviana* husks and fruits [26,41,42,43,44,45] is constituted by flavonoids. The chromatograms for the nine flavonoids found in the *P. peruviana* samples in this study are represented in Figure 16 and summarized in Table S1 (Supplementary Materials).

Table 3 summarizes the characteristic fragmentation patterns for these compounds, consisting of the loss of the glycoside to yield the aglycone, while other observed fragments corresponded to the rupture of the flavonoid ring [26,46,47].

**Figure 17:**
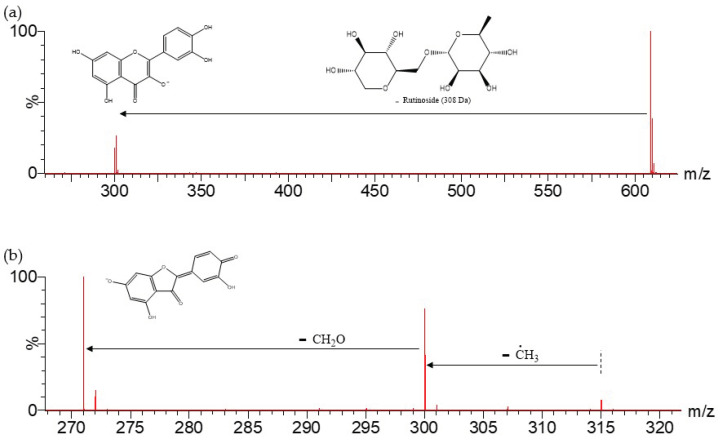
MS^2^ spectra for (**a**) compound 9, [M-H]^−^ = 609.1442, and (**b**) compound 31, [M-H]^−^ = 315.0504.

For instance, compound 9 ([M-H]^−^ = 609.1442), tentatively assigned to rutin, exhibited a main fragment at *m*/*z* 301 from the loss of the glycoside to yield quercetin, the corresponding aglycone [46] (Figure 17a). Meanwhile, compound 31 ([M-H]^−^ = 315.0504), tentatively assigned to isorhamnetin, presented a ring cleavage fragmentation pattern, yielding fragments at *m*/*z* 300 and 271 consistent with previous reports [47,48] (Figure 17b).

**Figure 18:**
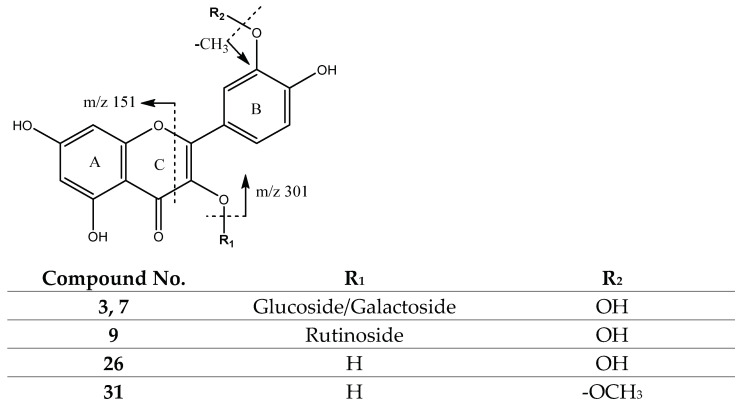
Quercetin derivatives structure and main fragments.

Aside from rutin (Rt = 16.78 min) (C_27_H_29_O_16_) [26,42], quercetin glycosides were present in peaks 3 (Rt = 12.60 min) and 7 (Rt = 16.08 min) with [M-H]^−^ at *m*/*z* 463.0894 (C_21_H_19_O_12_), tentatively assigned to quercetin O-glucoside/galactoside isomers [44], yielding their main fragments at *m*/*z* 301, corresponding to the aglycone (Figure 18). Meanwhile, aside from isorhamnetin (Rt = 24.16 min) (C_16_H_11_O_7_) [26] with fragments at *m*/*z* 300 and 271, peak 26 (Rt = 23.53 min) with [M-H]^−^ at 301.0362 (C_15_H_9_O_7_) was tentatively identified as quercetin, with its main fragments at *m*/*z* 151 due to Retro Diels–Alder on C-ring, and *m*/*z* 243 due to the loss of C_3_H_4_O_2_ from the A-ring [47] (Figure 18).

**Figure 19:**
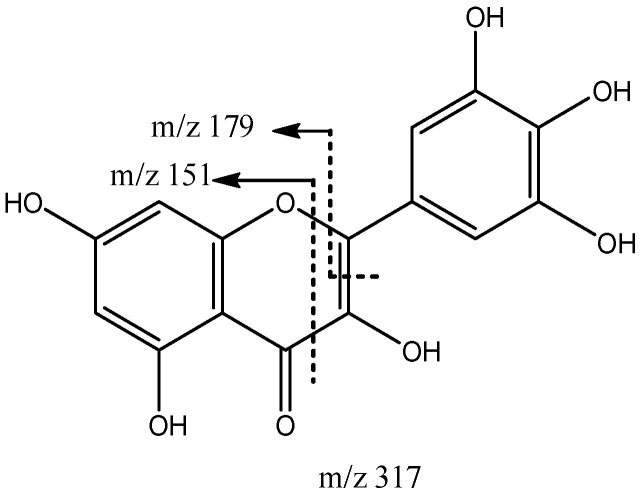
Myricetin fragmentation.

In turn, peak 5 (Rt = 15.35 min) with [M-H]^−^ at *m*/*z* 317.0305 (C_15_H_9_O_8_) was tentatively assigned to myricetin [42], with its main fragments at *m*/*z* 179 from retro-cyclization and *m*/*z* 151 due to retro Diels–Alder fragmentation, as previously reported [49] (Figure 19).

**Figure 20:**
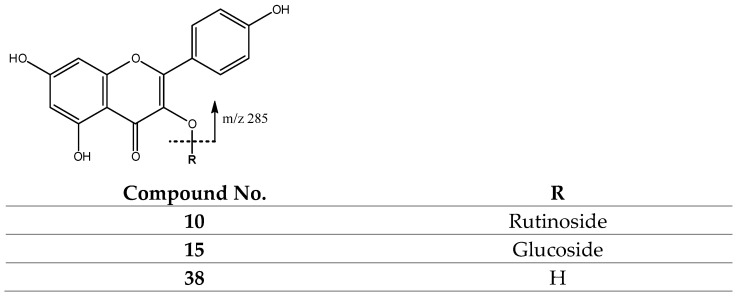
Kaempferol and derivatives’ structure and fragmentation.

Finally, peak 38 (Rt = 26.01 min) with [M-H]^−^ at *m*/*z* at 285.0410 (C_15_H_9_O_6_) was tentatively assigned to kaempferol [41], with its main fragments at *m*/*z* 257, 255 and 243, consistent with previous reports [50], and peaks 10 (Rt = 17.84 min) with [M-H]^−^ at *m*/*z* 593.1504 (C_27_H_29_O_15_) and 15 (Rt = 18.77 min) with [M-H]^−^ at 447.0947 (C_21_H_19_O_11_), which were tentatively assigned to kaempferol rutinoside [45] and kaempferol glucoside [26], respectively, both yielding a main fragment at *m*/*z* 285, corresponding to the aglycone (Figure 20).

**Figure 21:**
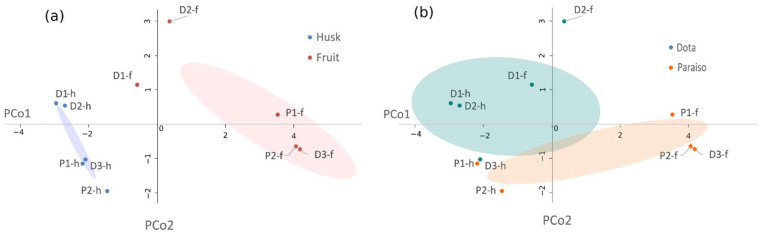
Principal coordinate plot performed over the Bray–Curtis distance matrix between the samples from the UPLC QTOF-ESI MS results. Ellipses show the mean distance to the centroid for each factor: (**a**) plant part and (**b**) region.

The UPLC-ESI-QTOF MS analysis results (summarized in Table S1) for husks and fruits (*n* = 10) show the D1 and D2 husks from the Dota region having a larger presence of the three types of secondary metabolites, while the P2 fruit had the lowest number of compounds. In order to further analyze the total characterization, accounting for 66 compounds distributed among all samples (*n* = 10), the Bray–Curtis distance matrix was obtained, and Figure 21 shows the principal coordinate analysis (PCoA) plot performed and the dispersion obtained from the data.

**Figure 22:**
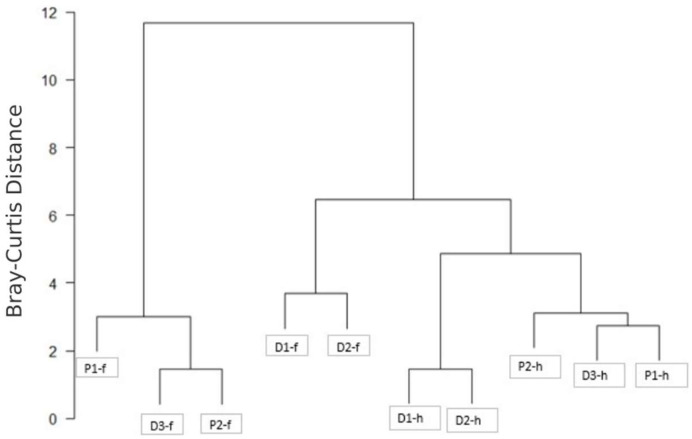
Hierarchical clustering analysis performed over the Bray–Curtis distance matrix between the samples from the UPLC-QTOF-ESI MS results.

When evaluating the dispersion of the distance matrix for both factors, no significant differences were found (*p* < 0.05) for the secondary metabolites’ diversity between the fruits and husks nor between the Dota and Paraiso regions. However, important differences could be asserted for the number of common peaks through hierarchical clustering (HC) analysis (Figure 22) in order to characterize the differences between samples due to the presence or absence of peaks detected by mass spectrometry [51].

In fact, from the dissimilarity matrix, the hierarchical clustering analysis shows three fruit samples, namely P1-f, D3-f and P2-f, forming a cluster. Meanwhile, the cluster distribution shows that the other two fruit samples, namely D1-f and D2-f from the Dota region, were closely related to their husk counterparts and belonged to the cluster represented by all five husk samples, therefore indicating that not necessarily all fruits are similar in compound composition among each other. Furthermore, within the husks, there were two groups: one constituted by P2-h, D3-h and P1-h, while D1-h and D2-h from the Dota region formed another group consisting of the samples with the highest number of compounds. Regarding regions, it is noteworthy that the samples from the Paraiso (P) were grouped or closely related within both husks and fruits, while the samples from the Dota (D) region were less homogeneous, with D3 grouped with the P samples for both parts. However, the distances among the P samples were larger than the distances between the D samples, which even when segregated into different clusters were closer to each other in terms of compound composition.

### 2.2. UPLC-DAD Analysis of Physalis peruviana Extracts

#### 2.2.1. Determination of β-Carotene in *P. peruviana* Fruits

UPLC-DAD analysis allowed quantification of the β-carotene present in the *Physalis peruviana* fruit extracts, following the extraction method and chromatographic protocol described in Section 2.2 and Section 2.3. The results are summarized in Table 4.

The results for the β-carotene content ranged from 43.00 μg/g of dry material to 60.10 μg/g of dry material, which was the highest value, corresponding to the D1-f sample. Recent reviews from the literature indicate that the β-carotene content in *P. peruviana fruits* is ≤2 mg/100 g fresh weight (FW) [52]. The results obtained in the present study align within this range, amounting to 0.68–1.43 mg/100 g FW. Previous reports showed variability in their results, such as studies in Peruvian fruits showing slightly higher values (1.77–2.64 mg/100 g FW) [53,54], while Costa Rican fruits showed better contents than the previously reported results for fruits from Colombia (0.14–0.31 mg/100 g FW) [55] and similar values to fruits from Argentina (1.24 mg/100 g FW) [56] and Egypt (1.53 mg/100 g FW) [41].

#### 2.2.2. Determination of Flavonoids in *P. peruviana* Fruits and Husks

UPLC-DAD analysis was performed on *P. peruviana* fruit and husk extracts obtained through PLE extraction under the protocols described in Section 2.2 and Section 2.3. The results are summarized in Table 5.

The results for the quercetin glucoside/galactoside (QG) content for the fruits ranged from 0.09 mg/g of dry material to 0.25 mg/g of dry material, which was the highest content, corresponding to the D1-f sample, followed by D2-f (0.15 mg/g of dry material). Compared with the literature, the results obtained were within the range of those reported for fruits from Egypt, showing 0.48 mg/g of dry material [41], and fruits from Brazil, with values ranging from 5.77 to 7.98 µg/g FW [42], compared with 19.04–26.24 µg/g FW in the present study.

In turn, the results for the UPLC-DAD analysis of the husks allowed the quantification of quercetin glucoside/galactoside (QG), rutin (RU) and quercetin (QC). The total quantified flavonoids ranged from 0.23 mg/g of dry material for P2-h to 2.83 mg/g of dry material for D2-h, representing the highest value, followed by D1-h (1.58 mg/g of dry material). Regarding rutin, the flavonoid with the more important content, D2-h also showed the greatest content (1.96 mg/g of dry material). The previously reported results for husk samples from Colombia indicated a rutin content ranging from 5.12 to 13.25 μg/mg of extract [43], while the samples from Costa Rica showed a higher content, ranging from 10.60 to 148.48 μg/mg of extract.

In order to assess the influence of the part used and the region of origin on the quantified flavonoids, two-way analysis of variance (ANOVA) was carried out for all samples (*n* = 10). The results showed a significant difference (*p* < 0.05) regarding both factors, with the samples from the Dota region yielding better results and the husks exhibiting a higher content. This result is in agreement with the findings from the literature accounting for a higher flavonoid content in husks in respect to fruits [44]. For instance, a study on Colombian goldenberry husks showed a flavonoid content of 195.4 mg/100 g of dry material [45], similar to the average of 189.84 mg/100 g of dry material for the Dota region samples in this study. In turn, the Colombian fruits exhibited a flavonoid content of 2.18 mg/100 g of dry material [45], which is lower than the average value of 14.1 mg/100 g of dry material for the Costa Rican fruits.

### 2.3. Folin–Ciocalteau Evaluation of P. peruviana Extracts

Recent studies [57,58] on different types of phenolic compounds have shown that the Folin–Ciocalteu assay, widely used to measure total polyphenolic contents, represents an adequate method for assessing the phenolic reducing capacity, occurring through a single electron transfer mechanism [59,60]. Table 6 summarizes the Folin–Ciocalteau (FC) results for extracts of *Physalis peruviana* fruits and husks, obtained through the PLE method as described in Section 2.4.

The FC results for the *P. peruviana* fruit extracts ranged from 1.51 mg gallic acid equivalents (GAE)/g of dry material to 2.60 mg GAE/g of dry material, which was the highest value, corresponding to the D1-f sample. Compared with the literature, the values obtained were similar to those previously reported for fruits from Chile (1.20–2.68 mg GAE/g of dry material) [61]. On the other hand, the values reported in terms of fresh weight for the fruits from Peru (15.20 mg GAE/100 g FW) [56], Chile (6.12–26.2 mg GAE/100 g FW) [62,63], Brazil (47.8–57.9 mg GAE/100 g of fresh weight) [64] and Colombia (60–140 mg GAE/100 g FW) [65] were within the range of or lower than the results for the Costa Rican fruits (34.5–59.4 mg GAE/100 g FW) in this study.

In turn, the FC results in the *P. peruviana* husk extracts presented values ranging from 0.84 mg GAE/g of dry material up to 5.12 mg GAE/g of dry material, which was the highest value, corresponding to sample D2-h. The FC values obtained were in agreement with the results previously reported for samples from Colombia, which ranged from 18.23 to 37.8 mg GAE/g of dry extract [66] and from 11.99 to 20.86 mg GAE/g of dry extract [43]. The samples from Cost Rica in this study showed similar and higher values, ranging from 6.74 to 36.44 mg GAE/g of dry extract.

In order to assess the influence of the part and the region of origin for the FC results, two-way ANOVA was carried out for all samples (*n* = 10). In a similar way to the results obtained for the quantification of flavonoids, the findings showed a significant difference (*p* < 0.05) for both factors, with the samples from the Dota region and husks exhibiting better results, which aligns with previous reports indicating higher FC values for husks in respect to fruits [45].

Finally, a Pearson correlation analysis was carried out between the FC values and the flavonoid quantification results. The findings showed a high positive correlation between the FC values with quercetin glucoside/galactosides (r = 0.926, *p* < 0.05) and rutin (r = 0.978, *p* < 0.05), which is in agreement with previous reports suggesting the influence of these metabolites on the FC results [67,68].

### 2.4. Evaluation of Antioxidant Activity of Physalis peruviana Extracts

The free radical scavenging activity can be evaluated using a reaction with a stable free radical such as 2,2-diphenyl-1-picrylhidrazyl (DPPH) [69]. Kinetic studies performed recently for this method have shown that the rate-determining step involves a fast electron transfer from phenoxide anions to DPPH, and therefore protic organic solvents enable this mechanism [70]. In order to perform the evaluation of the antioxidant activity of *P. peruviana* fruit and husk extracts, the DPPH method was applied as described in Section 3 of Materials and Methods. The results are summarized in Table 7.

The results for the antioxidant activity assays indicate samples D1-f and D2-f yielded the lowest IC_50_ values of 2.60 mg dry material/mL and 3.20 mg dry material/mL, respectively, therefore exhibiting better antioxidant activity. These observations are consistent with the results obtained for the FC total polyphenolic contents and flavonoid quantification. Compared with the literature, the results obtained for the *P. peruviana* fruit extracts showed better antioxidant values than the fruits from Colombia (IC_50_ = 5–15 mg of dry material/mL) [65] and from Portugal (IC_50_ of 81.5 mg FW/mL) [71], since the fruits from Costa Rica showed IC_50_ values ranging from 11.4 to 27.5 mg FW/mL.

In turn, considering the DPPH evaluation for Trolox (IC_50_ = 5.62 μg/mL) as a control, the Costa Rican fruit values (21.6–49.6 mg TE/100 g FW) were within the range of reports for fruits from Peru (24.9 mg TE/100 g FW) [53]. On the other hand, fruits from Turkey showed better results (11.49–16.47 μmol TE/g of dry material) [72], while the samples from Costa Rica held higher values (5.48–8.67 μmol TE/g of dry material) than the fruits from Chile (0.94–2.10 μmol TE/g of dry material) [61] and similar values to fruits from Poland (4.61–6.05 μmol TE/g of dry material) [73].

The results for the DPPH antioxidant activity evaluation of the husk extracts showed samples D2-h and D1-h yielding the lowest values of 1.62 mg dry material/mL and 2.25 mg dry material/mL, respectively, and therefore better antioxidant activity. Previous studies on husks from Colombia showed results varying from low antioxidant activity (0.764 µmol TE/g extract) [74] to higher values (4.94 mmol TE/g of dry material) [45]. Hence, considering Trolox was used as a control in this study, the husks from Costa Rica showed intermediate values of 41.28 µmol TE/g extract and 1.45 mmol TE/100 g of dry material.

In order to assess the influence of the part and the region of origin on DPPH antioxidant activity, two-way ANOVA was carried out for all samples (*n* = 10), with the results showing a significant difference (*p* < 0.05) for the regions, as the samples from the Dota region exhibited higher antioxidant activity with an average IC_50_ of 2.35 mg/mL. In turn, the results from a Pearson correlation analysis between the DPPH values and FC findings indicated a high negative correlation (r = −0.902, *p* < 0.05) among these variables, aligning with previous studies reporting correlation between the total polyphenolic content and antioxidant activity [75,76]. Furthermore, the Pearson correlation analysis, when performed separately for the fruit (*n* = 5) and husk samples (*n* = 5), showed a stronger negative correlation between the DPPH and FC values for the husks (r = −0.990, *p* < 0.05) and also a strong negative correlation between the DPPH and flavonoid quantification (r = −0.950, *p <* 0.05). These results suggest the important role that flavonoids play in contributing to the DPPH antioxidant activity in husks [76] and also align with studies suggesting that the role of β-carotene could impact the results in fruits [77].

**Figure 23:**
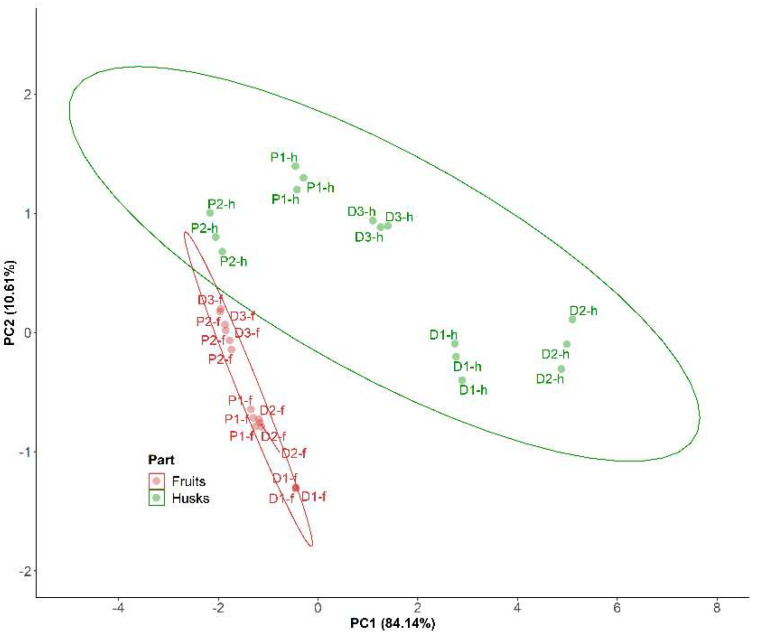
Plane defined by two first principal components (PC1 and PC2) resulting from PCA analysis of *P. peruviana* sample (*n* = 10). Regions: Dota (D) and Paraiso (P). Parts: husks (h) and fruits (f).

In order to summarize the results relating to the antioxidant evaluation, principal component analysis (PCA) was performed in all samples (*n* = 10), considering as variables the FC results and QG, QC, RU and DPPH values. Two components (PC1 and PC2) were obtained (loadings > 0.41), as shown in Figure 23. The first component (PC1) represented 84.64% of the total variance and showed positive correlation with the FC, QG and RU values. The second component (PC2) accounted for 10.58% of the total variance and was positively correlated with the QC and DPPH values.

**Figure 24:**
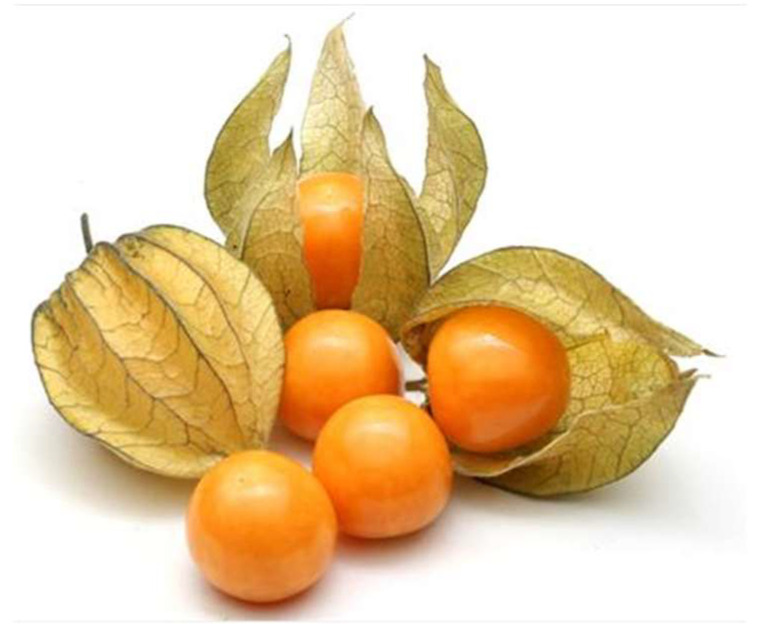
*P. peruviana* (Cape gooseberry) fruits with husks as commercialized by local producers from the Dota and Paraiso regions in Costa Rica.

As illustrated in the plane represented by the two components (Figure 24), the husks are distributed along PC1 and PC2, while the fruits show a less important distribution along PC1, with very low values indicating lower QG and RU flavonoid contents and lower FC total polyphenolic contents. In turn, the husks show high variability with the D1-h and D2-h samples, exhibiting particularly high values in PC1, which means higher RU and QG contents and better FC values. Furthermore, these two samples from the Dota region along with their corresponding fruits exhibited lower PC2 values, corresponding to higher DPPH antioxidant activity.

## 3. Materials and Methods

### 3.1. Physalis peruviana Samples, Chemicals and Reagents

*Physalis peruviana* fruits commercialized with their husks (Figure 24) were purchased in a ripe state from local producers in two regions of Costa Rica, namely the Santa Maria de Dota region (D1–D3) and the Paraiso region (P1 and P2). Solvents of an ACS or HPLC grade such as acetonitrile, ethyl acetate, ethanol and methanol were purchased from Baker (Center Valley, PA, USA). Reagents such as quercetin, rutin, β-carotene, sodium molibdate, gallic acid, 2,2-diphenyl-1-picrylhidrazyl (DPPH) and sodium tungstate were provided by Sigma-Aldrich (St. Louis, MO, USA).

### 3.2. Extraction of P. Peruviana Samples

All 10 samples (fruits and husks) were freeze-dried in a Free Zone −105 °C, 4.5-L Cascade Benchtop Freeze Dry System (Labconco, Kansas, MO, USA). The dried material was preserved in closed containers at −20 °C. Extractions for characterization of the secondary metabolites and quantification of the flavonoids were carried out in a Dionex™ASE™300 Accelerated Solvent Extractor (Thermo Scientific™, Walthman, MA, USA) using a previously reported method [26] with slight modifications. Briefly, 1 g of dry material was extracted using a mixture of ethanol:ethyl acetate (75:25) as a solvent at a temperature of 125 °C, and one cycle of 20 min of static time was applied. After extraction, the volume was completed at 10 mL with the solvent mixture.

In turn, extraction for the quantification of β-carotene in fruits (*n* = 5) was performed using 1.0 g of a freeze-dried sample that was mixed with 1 mL of a 12-mM MgCO_3_ solution and 5 mL of ethyl acetate and vortexed for 5 min at 3200 rpm in a Thermo Vortex Model 88,880,017 (Thermo Scientific, Waltham, MA, USA). After being kept at 5 °C for 15 min, the sample was vortexed for another 5 min at 3200 rpm and then centrifuged for 5 min at 4000 rpm in a Thermo Sorvall model ST-8 instrument (Thermo Scientific, Waltham, USA), and the supernatant was filtered to obtain the extract.

### 3.3. Analysis of P. Peruviana Extracts by UPLC-ESI-MS and by UPLC-DAD

To characterize *P. peruviana*‘s secondary metabolites, measurements were performed using a Xevo G2-XS QTOF coupled with an AQUITY H Class UPLC system with a quaternary pump (Waters, Wilmslow, UK) by adapting a previously reported method [26]. The ESI source parameters were set to a capillary voltage of 2 kV, sampling cone of 20 eV, source temperature of 150 °C, source offset of 10 °C, desolvation temperature of 450 °C, cone gas flow 0 L/h and desolvation gas flow of 900 L/h. The measurements were performed in MSe high-resolution negative mode using an acquisition mass range from 100 *m*/*z* to 1000 *m*/*z* and a scan rate of 0.5 s, where fragmentation was carried out using independent data acquisition for all eluting compounds, with a collision energy ramp from 20 V to 30 V stored at the high energy function. Instrument calibration was performed in the mass range of the measurements with sodium formate. Lock mass correction was applied directly to the measurements using leucine enkephalin infusion measured each 30 s during the run. The data were analyzed using MassLynx V4.2 software (Waters, Wilmslow, UK). A 1-µL sample was injected with a flow of 0.4 mL/min using a Luna RP-18 column (150 × 4.6 mm × 4 μm (Phenomenex Inc., Torrance, CA, USA)) at 30 °C and a chromatographic gradient starting at 0% B and increasing to 70% B at 15 min, 80% B at 35 min and then 100% B at 38 min, at which point the gradient was held for 6 min, and then the column was equilibrated for 5 min to the initial conditions. The solvents used in the mobile phase were A (water) with 0.1% formic acid and B (acetonitrile) with 0.1% formic acid.

In turn, a Thermo PDA eλ photodiode array detector coupled with a Thermo Scientific Dionex UltiMate 3000 UPLC system (Thermo Scientific, Waltham, MA, USA) was used to develop a method for quantification of the main flavonoids in the fruit and husk extracts (*n* = 10). Rutin and quercetin were used as standards, and their calibration curves were prepared. A Phenomenex® RP-18 column (2.1 × 150 mm; 4.6 μm) was used at 30 °C and a flow rate of 0.4 mL/min. Acetonitrile and water acidified with formic acid were used as solvents. The gradient consisted of solvent A, water:formic acid (0.1%), and solvent B, acetonitrile:formic acid (0.1%), and was applied as follows: 0% B at 0 min, 30% B at 15 min, 67.5% B at 30 min, 100% B at 31–35 min, 0% B at 36 min and 0% B at 42 min.

Finally, an Agilent PDA eλ photodiode array detector coupled with an Agilent 1260 HPLC system (Agilent Scientific Instruments, Santa Clara, CA, USA) was used to develop a method for quantification of the β-carotene in the different fruit extracts (*n* = 5), and previously, a β- carotene calibration curve was prepared. A Phenomenex® RP-18 column (2.1 × 150 mm; 4.6 μm) (Phenomenex Inc, Torrence, CA, USA) was used at 30 °C and a flow rate of 1 mL/min. The gradient consisted of solvent A, methanol (MeOH), solvent B, acetonitrile (MeCN), and solvent C, isopropanol (IPA), and it was applied as follows: 40% B and 40% C at 0 min, 40% B and 60% C at 15 min and 40% B and 40% C at 16–20 min.

### 3.4. Determination of Folin–Ciocalteu Total Polyphenolic Contents

All extracts were evaluated through a variation of the Singleton and Rossi method using the Folin–Ciocalteu (FC) reagent, which consists of a mixture of phosphomolybdic and phosphotungstic acids. As previously described [78], the method consists of mixing 0.5 mL of FC reagent and 10 mL of Na_2_CO_3_ (7.5%) with 0.5 mL of *P. peruviana* husk or fruit extract dissolved in acidified MeOH (0.1% HCl) to assure extract dissolution regardless of the solvent used for plant material extraction. Finally, water is added for a complete 25-mL volume. A blank was prepared similarly by using 0.5 mL of MeOH (0.1% HCl) instead of the extract. Both the blank and extract mixtures were left standing in the dark for 1 h, and the subsequent absorbance was measured at 750 nm. The absorbance values were extrapolated in a gallic acid calibration curve that was formed in order to obtain the FC results, which are expressed as mg gallic acid equivalents (GAE)/g of dry material. Analyses were performed in triplicate.

### 3.5. DPPH Radical Scavenging Activity

DPPH evaluation was performed as previously reported [79]. Briefly, a solution of 2,2-diphenyl-1-picrylhidrazyl (DPPH) (0.25 mM) was prepared using methanol as a solvent. Next, 0.5 mL of this solution was mixed with 1 mL of *P. peruviana* fruit or husk extract at different concentrations. These solutions were incubated at 25 °C in the dark for 30 min. DPPH absorbance was measured at 517 nm. Blanks were prepared for each concentration. Trolox was used as a control. The inhibition percentage was determined as shown in the following equation:

$$\mathrm{Inhibition}\;\mathrm{percentage}\;\left(\%\right)=\frac{\left({\mathrm{Abs}}_{\mathrm{blank}}-{\mathrm{Abs}}_{\mathrm{sample}}\right)}{{\mathrm{Abs}}_{\mathrm{blank}}}\times 100$$

The inhibition percentage was plotted against the respective sample concentration to determine the IC_50_, which corresponds to the quantity of the sample required to reach 50% radical scavenging activity. Each sample was analyzed in three independent assays.

### 3.6. Statistical Analysis

Principal coordinates analysis (PCoA), hierarchical clustering analysis (HC) and a permutation test were performed on the Bray Curtis dissimilarity matrix generated from the identified compounds through UPLC QTOF-ESI MS. One-way analysis of variance (ANOVA) with a Tukey post hoc test as statistical tests were applied to the flavonoid, β-carotene, FC and DPPH measurements to determine any significant differences (*p* < 0.05) between samples. Two-way ANOVA was applied to evaluate both factors (i.e., *P. peruviana* parts and the regions of origin). Pearson correlation analysis was applied to the flavonoid contents measured by UPLC-DAD, FC total polyphenolic contents and DPPH antioxidant activity results. Finally, principal component analysis (PCA) was applied to summarize the data from the antioxidant activity results. R (version 1.2.1335) was used as the statistical program.

## 4. Conclusions

In summation, the findings for the *P. peruviana* husks and fruits clearly indicate compositions analogous to the previously reported results for cape gooseberry material, while the secondary metabolites’ diversity was similar among the fruits and husks and also for the location of origin. However, not all of the fruits were homogeneous in composition within them, and two fruit samples from the Dota region belonged to the husks cluster. Our results also indicate better antioxidant activity for the husk counterparts of those two fruit samples from the Dota region, thus showing their potential for further studies.

Considering the diverse functionalities exerted by the different secondary metabolites found in *P. peruviana* fruits and husks, the possibility of using these naturally occurring compounds can offer a future alternative strategy for the prevention and treatment of diseases with functional food. Further research and toxicological and pharmacological data are needed in order to support future innovative therapeutic approaches and commercialization possibilities to strengthen these fruits’ value chain.

## Figures and Tables

**Table 1:** UPLC-ESI-MS characterization of withanolides in *Physalis peruviana* samples from Costa Rica.

Peak No.	[M-H]^−^	Lactone Moiety (Da)	[Precursor] -Lac (*m*/*z*)	Other Fragments
4	543.2612	124	[543]: 419	483[543-HOAc]^−^,359[483-Lac]^−^, 341[359-H_2_O]^−^, 323 [341-H_2_O]^−^
6, 17, 24	517.2471	140	[517]: 377	499[517-H_2_O]^−^, 359[377-H_2_O]^−^, 341[359-H_2_O]^−^
12, 14, 18	521.2756	142	[521]: 379	503[521-H_2_O]^−^, 361[379-H_2_O]^−^, 343[361-H_2_O]^−^
13	519.2601	140	[519]: 379	501[519-H_2_O]^−^, 361[379-H_2_O]^−^, 343[361-H_2_O]^−^
20, 25	519.2601	142	[519]: 377	501[519-H_2_O]^−^, 359[377-H_2_O]^−^, 341[359-H_2_O]^−^
21, 35	547.2926	124	[465]: 341	501[547-EtOH]^−^, 483[501-H_2_O]^−^, 465[483-H_2_O]^−^
22	501.2494	140	[501]: 361	483[501-H_2_O]^−^, 343[361-H_2_O]^−^, 325[341-H_2_O]^−^
23, 30	661.3590	124	[481]: 357	601[647-HOAc]^−^, 481[661-OGluc]^−^, 339[357-H_2_O]^−^
27,33	501.2494	124	[501]: 377	483[501-H_2_O]^−^, 359[377-H_2_O]^−^, 341[359-H_2_O]^−^
28	631.3137	124	[433]: 309	631[613-H_2_O]^−^, 451[631-OGluc]^−^, 433[451-H_2_O]^−^
29, 34	503.2650	142	[503]: 361	485[503-H_2_O]^−^, 343[361-H_2_O]^−^, 325[343-H_2_O]^−^
36, 40, 42	485.2550	124	[485]: 361	467[485-H_2_O]^−^, 343[361-H_2_O]^−^
37, 43, 45	487.2701	142	[487]: 345	469[487-H_2_O]^−^, 327[345-H_2_O]^−^
41, 44	471.2725	158	[471]: 313	453[471-H_2_O]^−^, 435[453-H_2_O]^−^
48	469.2607	124	[469]: 345	451[469-H_2_O]^−^, 327 [345-H_2_O]^−^
50, 51	513.2831	144	[453]: 309	495[513-H_2_O]^−^, 453[513-HOAc]^−^
54, 55	561.3042	144	[471]: 327	531[561-CH_2_O]^−^, 471[531-HOAc]^−^, 453[471-H_2_O]^−^
57	649.3606	140	[469]: 329	631[649-H_2_O]^−^, 469[649-OGluc]^−^,

**Table 2:** UPLC-ESI-MS characterization of sucrose esters in *P. peruviana* samples from Costa Rica.

Peak No.	[M-H +FA]^−^	[M-H]^−^ (*m*/*z*)	R group (Da)	[M-H-R]^−^ (*m*/*z*)	Other Fragments (*m*/*z*)
1, 2	457.1558	411	70	341	323[Suc-H-H_2_O]^−^, 181[Suc-H-Gluc]^−^
8, 11	527.1980	481	70	411	393[411-H_2_O]^−^, 323[Suc-H-H_2_O]^−^
16	541.2151	495	84	411	393[411-H_2_O]^−^, 323[Suc-H-H_2_O]^−^, 161[Suc-H-OGluc]^−^
19	609.2408	563	82	481	411[481-70]^−^, 393[411-H_2_O]^−^
32	597.2380	551	70	481	411[481-70]^−^, 393[411-H_2_O]^−^
39	623.2604	577	84	493	411[493-82]^−^, 323[Suc-H-H_2_O]^−^
46	611.2938	565	154	411	495[565-70]^−^, 323[Suc-H-H_2_O]^−^
47	653.3061	607	126	481	537[607-70]^−^, 393[481-70-H_2_O]^−^
49	723.3472	677	126	551	607[677-70]^−^, 481[551-70]^−^
52, 58, 62	693.3303	647	82	565	493 [647-154]^−^, 411[493-82]^−^
53	667.3208	621	140	481	551[621-70]^−^, 411 [481-70]^−^
56, 59	681.3366	635	154	481	411 [481-70]^−^, 393[411-H_2_O]^−^
60, 61	737.3571	691	140	551	621[691-70]^−^, 481 [551-70]^−^, 393[481-70-H_2_O]^−^
63	751.3787	705	154	551	635[705-70]^−^, 481 [551-70]^−^, 393[481-70-H_2_O]^−^
64	765.3963	719	154	565	635[719-84]^−^, 481 [565-84]^−^, 393[481-70-H_2_O]^−^
65	793.4285	747	182	565	663[747-84]^−^, 481 [565-84]^−^, 393[481-70-H_2_O]^−^
66	779.4506	733	182	551	593[733-140]^−^, 411 [481-70]^−^

**Table 3:** UPLC-ESI-MS characterization of flavonoids in *Physalis peruviana* samples from Costa Rica.

Peak No.	[M-H]^−^	Glycoside group (Da)	Aglycone (*m*/*z*)	Other Fragments (*m*/*z*)
3,7	463.0894	Glucose/Galactose (162)	301	
5	317.0305		317	151, 179
9	609.1442	Rutinoside (308)	301	
10	593.1504	Rutinoside (308)	285	
15	447.0947	Glucose/Galactose (162)	285	
26	301.0362		301	243, 163, 151
31	315.0504		315	300, 271
38	285.0410		285	257, 255, 243

**Table 4:** β-carotene content for fruit extracts of *Physalis peruviana*.

Product	β-Carotene (μg/g) ^1,2,3^
D1-f	60.10 ^a^ ± 0.08
D2-f	53.67 ^d^ ± 0.09
D3-f	58.60 ^b^ ± 0.38
P1-f	55.90 ^c^ ± 1.01
P2-f	43.00 ^e^ ± 0.21

^1^ μg/g of dry material. ^2^ Values are expressed as mean ± standard deviation (SD). ^3^ Different superscript letters indicate differences are significant at *p* < 0.05 using one-way analysis of variance (ANOVA) with a Tukey post hoc test as a statistical test.

**Table 5:** Flavonoid content for fruit and husk extracts of *Physalis peruviana*.

Product ^1^	Quercetin Glucoside/Galactoside (QG) (mg/g) ^2,3,4^	Product ^1^	Quercetin Glucoside/Galactoside (QG) (mg/g) ^2,3,4^	Quercetin (QC) (mg/g) ^2,^^3,4^	Rutin (RU) (mg/g) ^2,3^^,4^
D1-f	0.25 ^a^ ± 0.02	D1-h	0.58 ^a^ ± 0.05	0.21 ^a^ ± 0.02	0.75 ^b^ ± 0.01
D2-f	0.15 ^b^ ± 0.01	D2-h	0.64 ^a^ ± 0.03	0.23 ^a^ ± 0.02	1.96 ^a^ ± 0.04
D3-f	0.09 ^d^ ± 0.00	D3-h	0.33 ^b^ ± 0.01	0.22 ^a^ ± 0.01	0.74 ^b^ ± 0.04
P1-f	0.11 ^c^ ± 0.01	P1-h	0.19 ^c^ ± 0.01	0.22 ^a^ ± 0.01	0.15 ^c^ ± 0.01
P2-f	0.10 ^cd^ ± 0.00	P2-h	0.04 ^d^ ± 0.00	0.05 ^b^ ± 0.00	0.14 ^c^ ± 0.01

^1^ Region: D = Dota and P = Paraiso. Part: h = husk. ^2^ Expressed as mg/g of dry material. ^3^ Values are expressed as mean ± standard deviation (SD). ^4^ Different superscript letters in the same column indicate differences are significant at *p* < 0.05 using one-way analysis of variance (ANOVA) with a Tukey post hoc test as a statistical test.

**Table 6:** Folin–Ciocalteau (FC) results for *P. peruviana* fruit and husk extracts.

Product	FC (mg GAE/g) ^1,2,3^	Product	FC (mg GAE/g) ^1,2,3^
D1-f	2.60 ^a^ ± 0.01	D1-h	4.28 ^b^ ± 0.08
D2-f	1.89 ^b^ ± 0.03	D2-h	5.12 ^a^ ± 0.12
D3-f	1.74 ^b^ ± 0.03	D3-h	2.59 ^c^ ± 0.07
P1-f	1.88 ^b^ ± 0.08	P1-h	1.14 ^d^ ± 0.01
P2-f	1.51 ^c^ ± 0.10	P2-h	0.84 ^e^ ± 0.01

^1^ Expressed as mg of gallic acid equivalent (GAE)/g of dry material. ^2^ Values are expressed as mean ± standard deviation (SD). ^3^ Different superscript letters in the same column indicate differences are significant at *p* < 0.05 using one-way analysis of variance (ANOVA) with a Tukey post hoc test as a statistical test.

**Table 7:** DPPH antioxidant activity of fruit and husk extracts from *P. peruviana*.

Product	IC_50_ (mg/mL) ^1,2,3^	Product	IC_50_ (mg/mL) ^1,2,3^
D1-f	2.60 ^e^ ± 0.01	D1-h	2.25 ^d^ ± 0.02
D2-f	3.20 ^d^ ± 0.04	D2-h	1.62 ^e^ ± 0.09
D3-f	4.49 ^a^ ± 0.21	D3-h	3.19 ^c^ ± 0.14
P1-f	3.24 ^c^ ± 0.10	P1-h	3.98 ^b^ ± 0.16
P2-f	4.33 ^b^ ± 0.23	P2-h	4.72 ^a^ ± 0.82

^1^ IC_50_ mg of dry material/mL. ^2^ Values are expressed as mean ± standard deviation (SD). ^3^ Different superscript letters in the same column indicate differences are significant at *p* < 0.05 using one-way analysis of variance (ANOVA) with a Tukey post hoc test as a statistical test.

## Data Availability

The data presented in this study are available within this article.

## Supplementary Materials

The following supporting information can be downloaded from https://www.mdpi.com/article/10.3390/molecules27134238/s1, Table S1: Compounds in *Physalis peruviana* husks and fruits from Dota and Paraiso regions in Costa Rica.

## Sample Availability

Samples of the compound are not available from the authors.
