# Supplementary material for: QTOF-ESI MS Characterization and Antioxidant Activity of Physalis peruviana L. (Cape Gooseberry) Husks and Fruits from Costa Rica
Source: Molecules. 2022 Jun 30;27(13):4238. doi: 10.3390/molecules27134238 (PMC9268663; doi:10.3390/molecules27134238)
Supplement: Supplementary file 1 [file molecules-27-04238-s001.zip › Table S1.pdf]

**Table S1.** Compounds in *Physalis peruviana* husks and fruits from Dota and Paraiso regions in Costa Rica.

| No. | Tentative identification | Rt (min) | Formula | $[M-H]^-$ | Error (ppm) | Husk[1] | Fruit[1] | Ref[2] |
|---|---|---|---|---|---|---|---|---|
| **1** | O-isobutanoylsucrose (isomer I of II) | 11.87 | $C_{16}H_{28}O_{12}$ | 411.1528 | 6.202 | D1, D2, D3 P1, P2 | D1, D2, D3 P1, P2 | 26 |
| **2** | O-isobutanoylsucrose (isomer II of II) | 12.43 | $C_{16}H_{28}O_{12}$ | 411.1528 | 6.202 | D1, D2, D3 P1, P2 | D1, D2, D3 | 26 |
| **3** | Quercetin-glucoside/ galactoside | 12.60 | $C_{21}H_{20}O_{12}$ | 463.0894 | 3.779 | D1, D2, D3 P1, P2 | D1, D2, D3 P1, P2 | 44 |
| **4** | Physagulin O | 14.91 | $C_{30}H_{40}O_{9}$ | 543.2612 | 3.295 | D1, D2 | D1, D2 | 32 |
| **5** | Myrecitin | 15.35 | $C_{15}H_{10}O_{8}$ | 317.0305 | 2.397 | D1, D2, D3 P1, P2 | D1, D2, D3 P1, P2 | 42 |
| **6** | 4β,27-hydroxywithanolide E (isomer I of III) | 15.68 | $C_{28}H_{38}O_{9}$ | 517.2471 | 6.457 | D1, D2, D3 P1, P2 | D1, D2, D3 P1, P2 | 26 |
| **7** | Quercetin-glucoside/ galactoside | 16.08 | $C_{21}H_{20}O_{12}$ | 463.0894 | 3.779 | D1, D2, D3 P1, P2 | | 44 |
| **8** | Di-O-isobutanoylsucrose (isomer I of II) | 16.71 | $C_{20}H_{34}O_{13}$ | 481.1960 | 8.063 | D1, D2, D3 P1, P2 | D1, D2 | 26 |
| **9** | Rutin | 16.78 | $C_{27}H_{30}O_{16}$ | 609.1442 | -2.233 | D1, D2, D3 P1, P2 | D1, D2, D3 P1, P2 | 43 |
| **10** | Kaempferol-rutinoside | 17.84 | $C_{27}H_{30}O_{15}$ | 593.1504 | -0.421 | D1, D2, D3 P1, P2 | | 45 |
| **11** | Di-O-isobutanoylsucrose (isomer II of II) | 17.72 | $C_{20}H_{34}O_{13}$ | 481.1960 | 8.063 | D1, D2, D3 P1, P2 | D1, D2 | 26 |
| **12** | 2,3,24,25-tetrahydro-4,27-dihydroxylated withanolide E (isomer I of III) | 17.88 | $C_{28}H_{42}O_{9}$ | 521.2756 | 1.036 | D1, D2, D3 P1, P2 | D1, D2 | 26 |
| **13** | 2,3-Dihydro-27-hydroxy-4β-hydroxywithanolide E (isomer I of III) | 18.28 | $C_{28}H_{40}O_{9}$ | 519.2601 | 1.329 | D1, D2, D3 P1, P2 | D1, D2, D3 P1, P2 | 26 |
| **14** | 2,3,24,25-tetrahydro-4,27-dihydroxylated withanolide E (isomer II of III) | 18.30 | $C_{28}H_{42}O_{9}$ | 521.2756 | 1.036 | D1, D2 P1 | D1, D2 P1 | 26 |
| **15** | Kaempferol glucoside | 18.77 | $C_{21}H_{20}O_{11}$ | 447.0947 | 4.384 | D1, D2, D3 P1, P2 | | 26 |
| **16** | o-isobutanoyl-o-(2-methylbutanoyl)sucrose | 19.17 | $C_{21}H_{36}O_{13}$ | 495.2117 | 7.936 | D1, D2, D3 P1, P2 | D1, D2 | 26 |
| **17** | 4β,27-hydroxywithanolide E (isomer II of III) | 19.60 | $C_{28}H_{38}O_{9}$ | 517.2471 | 6.457 | D2, D3 P1, P2 | D1, D2 | 26 |
| **18** | 2,3,24,25-tetrahydro-4,27-dihydroxylated withanolide E (isomer III of III) | 20.22 | $C_{28}H_{42}O_{9}$ | 521.2756 | 1.036 | D1, D2, D3 P1 | D1, D2 P1 | 26 |
| **19** | di-o-isobutanoyl-o-pentenoylsucrose | 21.04 | $C_{25}H_{40}O_{14}$ | 563.2372 | 5.717 | D1, D2, D3 P1, P2 | | 26 |
| **20** | 24,25-Dihydro-4,27-dihydroxylated withanolide E (isomer II of III) | 22.28 | $C_{28}H_{40}O_{9}$ | 519.2601 | 1.329 | D1, D2, D3 P1 | D1, D3 | 26 |
| **21** | Phyperunolide F (isomer I of II) | 22.33 | $C_{30}H_{44}O_{9}$ | 547.2926 | 3.453 | D1, D2, D3 P1 | | 38 |
| **22** | 17,27-dihydroxylated withanolide D | 22.38 | $C_{28}H_{38}O_{8}$ | 501.2494 | 1.117 | D1, D2, D3 P1, P2 | D1, D3 | 26 |

| No. | Tentative identification | Rt (min) | Formula | [M-H]- | Error (ppm) | Husk[1] | Fruit[1] | Ref[2] |
|---|---|---|---|---|---|---|---|---|
| **23** | Physalolactone B-3-O-β-glucopyranoside (isomer I of II) | 22.51 | $C_{36}H_{54}O_{11}$ | 661.3590 | 0.318 | D1, D2, D3 | D2<br>P1 | 32 |
| **24** | 4β,27-hydroxywithanolide E (isomer III of III) | 23.01 | $C_{28}H_{38}O_{9}$ | 517.2471 | 6.457 | D1, D2, D3<br>P1, P2 | D1, D2 | 26 |
| **25** | 24,25-Dihydro-4,27-dihydroxylated withanolide E (isomer III of III) | 23.19 | $C_{28}H_{40}O_{9}$ | 519.2601 | 1.329 | D1, D2, D3<br>P2 | D1, D2<br>P1 | 26 |
| **26** | Quercetin | 23.53 | $C_{15}H_{10}O_{7}$ | 301.0362 | 4.551 | D1, D2, D3<br>P1 | D1, D2 | 42 |
| **27** | 4β-hydroxywithanolide E (isomer I of II) | 23.57 | $C_{28}H_{38}O_{8}$ | 501.2494 | 1.117 | D1, D2, D3<br>P1, P2 | D1, D2, D3<br>P1, P2 | 26 |
| **28** | 17ß-hydroxy-14,20-epoxy-1-oxo-[22R]-3 ß -[O-ß-D-glucopyranosyl]-witha-5,24-dienolide | 23.59 | $C_{34}H_{48}O_{11}$ | 631.3137 | 2.946 | D1, D2, D3 | | 39 |
| **29** | 24,25-dihydro-17,27-dihydroxylated withanolide D (isomer I of II) | 23.64 | $C_{28}H_{40}O_{8}$ | 503.2650 | 1.013 | D1, D2, D3<br>P2 | D1 | 26 |
| **30** | Physalolactone B-3-O-β-glucopyranoside (isomer II of II) | 23.66 | $C_{36}H_{54}O_{11}$ | 661.3590 | 0.318 | D1, D2 | D2 | 32 |
| **31** | Isorhamnetin | 24.16 | $C_{16}H_{12}O_{7}$ | 315.0504 | -0.254 | D2, D3<br>P1, P2 | D1, D2 | 26 |
| **32** | o-tri-o-isobutanoylsucrose | 24.42 | $C_{24}H_{40}O_{14}$ | 551.2330 | -1.778 | D1, D2, D3<br>P1, P2 | D1, D2, D3<br>P1, P2 | 26 |
| **33** | 4β-hydroxywithanolide E (isomer II of II) | 24.56 | $C_{28}H_{38}O_{8}$ | 501.2494 | 1.117 | D1, D2, D3<br>P1, P2 | D1 | 26 |
| **34** | 24,25-dihydro-17,24-dihydroxylated withanolide D (isomer II of II) | 24.72 | $C_{28}H_{40}O_{8}$ | 503.2650 | 1.013 | D1, D2, D3<br>P1, P2 | D1, D2 | 26 |
| **35** | Phyperunolide F (isomer II of II) | 25.38 | $C_{30}H_{44}O_{9}$ | 547.2926 | 3.453 | D1, D2, D3<br>P1, P2 | D1 | 38 |
| **36** | Withanolide E (isomer I of III) | 25.89 | $C_{28}H_{38}O_{7}$ | 485.2550 | 2.205 | D1, D2, D3<br>P1, P2 | D1, D2 | 26 |
| **37** | 24,25-dihydro-24-hydroxylated withanolide D (isomer I of III) | 25.93 | $C_{28}H_{40}O_{7}$ | 487.2701 | 1.067 | D1, D2, D3<br>P1, P2 | D1 | 26 |
| **38** | Kaempferol | 26.01 | $C_{15}H_{10}O_{6}$ | 285.0410 | 3.824 | D1, D2 | | 41 |
| **39** | o-isobutanoyl-o-(2-methylbutanoyl)-o-pentenoylsucrose | 26.06 | $C_{26}H_{42}O_{14}$ | 577.2532 | 6.184 | D1, D2, D3<br>P1, P2 | D1, D2 | 26 |
| **40** | Withanolide E (isomer II of III) | 26.55 | $C_{28}H_{38}O_{7}$ | 485.2550 | 2.205 | D1, D2, D3<br>P1, P2 | D1, D2 | 26 |
| **41** | Peruvianolide D (isomer I of II) | 27.81 | $C_{28}H_{40}O_{6}$ | 471.2725 | -4.583 | D1, D2, D3<br>P1, P2 | D1 | 36 |
| **42** | Withanolide E (isomer III of III) | 28.15 | $C_{28}H_{38}O_{7}$ | 485.2550 | 2.205 | D1, D2, D3<br>P1, P2 | D1, D2 | 26 |
| **43** | 24,25-dihydro-24-hydroxylated withanolide D (isomer II of III) | 28.94 | $C_{28}H_{40}O_{7}$ | 487.2701 | 1.067 | D1, D2, D3<br>P1, P2 | | 26 |
| **44** | Peruvianolide D (isomer II of II) | 29.67 | $C_{28}H_{40}O_{6}$ | 471.2725 | -4.583 | D1, D2 | D2 | 36 |
| **45** | 24,25-dihydro-24-hydroxylated withanolide D (isomer III of III) | 30.18 | $C_{28}H_{40}O_{7}$ | 487.2701 | 1.067 | D1, D2, D3<br>P1, P2 | | 26 |

| No. | Tentative identification | Rt (min) | Formula | [M-H]⁻ | Error (ppm) | Husk[1] | Fruit[1] | Ref[2] |
|---|---|---|---|---|---|---|---|---|
| **46** | o-decanoyl-o-isobutanoylsucrose | 30.21 | $C_{26}H_{46}O_{13}$ | 565.2908 | 8.456 | D1, D2, D3 P1, P2 | D1, D2 | 26 |
| **47** | Di-O-isobutanoyl-O-octanoylsucrose | 31.02 | $C_{28}H_{48}O_{14}$ | 607.3018 | 8.595 | D1, D2, D3 P1, P2 | D1 | 26 |
| **48** | Withanolide D | 31.19 | $C_{28}H_{38}O_6$ | 469.2607 | 3.601 | D1, D2, D3 P1, P2 | | 26 |
| **49** | o-octanoyl-tri-o-isobutanoylsucrose | 32.13 | $C_{32}H_{54}O_{15}$ | 677.3428 | 6.422 | D1, D2, D3 P1, P2 | D1, D2, D3 P1, P2 | 31 |
| **50** | Virginol C (isomer I of II) | 35.02 | $C_{30}H_{42}O_7$ | 513.2831 | -4.150 | D1, D2, D3 P1 | D1, D2 | 37 |
| **51** | Virginol C (isomer II of II) | 35.66 | $C_{30}H_{42}O_7$ | 513.2831 | -4.150 | D1, D2, P1 | D1, D2 | 37 |
| **52** | o-decanoyl-o-isobutanoyl-o-(2-methylbutenoyl)sucrose (isomer I of III) | 36.84 | $C_{31}H_{52}O_{14}$ | 647.3217 | -9.547 | D1, D2, D3 P1, P2 | | 26 |
| **53** | di-o-isobutanoyl-o-nonanoylsucrose | 37.18 | $C_{29}H_{50}O_{14}$ | 621.3139 | 2.688 | D1, D2, D3, P1, P2 | D1, D2 P1 | 26 |
| **54** | Peruvianolide E (isomer I of II) | 37.65 | $C_{31}H_{46}O_9$ | 561.3042 | -3.848 | D1, D2 | D2 | 36 |
| **55** | Peruvianolide E (isomer II of II) | 38.31 | $C_{31}H_{46}O_9$ | 561.3042 | -3.848 | D1, D2 | D2 | 36 |
| **56** | di-o-isobutanoyl-o-decanoylsucrose (isomer I of II) | 38.37 | $C_{30}H_{52}O_{14}$ | 635.3285 | 0.976 | D1, D2, D3 P1, P2 | D1, D2, D3 P1, P2 | 26 |
| **57** | Daturafoliside B | 39.17 | $C_{35}H_{54}O_{11}$ | 649.3606 | 2.787 | D1, D2, D3 P1, P2 | D1, D2, D3 P1, P2 | 40 |
| **58** | o-decanoyl-o-isobutanoyl-o-(2-methylbutenoyl)sucrose (isomer II of III) | 39.98 | $C_{31}H_{52}O_{14}$ | 647.3217 | -9.547 | D1, D2, D3 P1, P2 | D1, D2 P1 | 26 |
| **59** | di-o-isobutanoyl-o-decanoylsucrose (isomer II of II) | 41.44 | $C_{30}H_{52}O_{14}$ | 635.3285 | 0.976 | D1, D2, D3 P1, P2 | D1, D2 | 26 |
| **60** | o-nonanoyl-tri-o-isobutanoylsucrose (isomer I of II) | 41.57 | $C_{33}H_{56}O_{15}$ | 691.3513 | -4.050 | D1, D2, D3 P1, P2 | D1, D2, D3 P1, P2 | 26 |
| **61** | o-nonanoyl-tri-o-isobutanoylsucrose (isomer II of II) | 42.11 | $C_{33}H_{56}O_{15}$ | 691.3513 | -4.050 | D1, D2, D3 P1, P2 | D1, D2, D3 P1, P2 | 26 |
| **62** | o-decanoyl-o-isobutanoyl-o-(2-methylbutenoyl)sucrose (isomer III of III) | 42.63 | $C_{31}H_{52}O_{14}$ | 647.3217 | -9.547 | D1, D2, D3 P1, P2 | D1, D2, D3 P1, P2 | 26 |
| **63** | O-decanoyl-tri-o-isobutanoylsucrose | 42.89 | $C_{34}H_{58}O_{15}$ | 705.3760 | 8.861 | D1, D2, D3 P1, P2 | D1, D2, D3 P1, P2 | 31 |
| **64** | di-o-isobutanoyl-o-decanoyl-o-(2-methylbutanoyl)sucrose | 43.85 | $C_{35}H_{60}O_{15}$ | 719.3883 | 4.031 | D1, D2, D3 P1, P2 | D1, D2, D3 P1, P2 | 31 |
| **65** | di-o-isobutanoyl-o-dodecanoyl-o-(2-methylbutanoyl)sucrose | 44.34 | $C_{37}H_{64}O_{15}$ | 747.4202 | 4.683 | D1, D2 P1, P2 | | 31 |
| **66** | o-dodecanoyl-o-isobutanoyl-o-nonanoylsucrose | 45.96 | $C_{37}H_{66}O_{14}$ | 733.4395 | 2.822 | D1, D2, D3 P1, P2 | | 26 |

[1] Region: D = Dota, P = Paraiso [2] Compound Report Reference.
